# Association of Fok1 VDR polymorphism with Vitamin D and its associated molecules in pulmonary tuberculosis patients and their household contacts

**DOI:** 10.1038/s41598-019-51803-8

**Published:** 2019-10-24

**Authors:** Sudhasini Panda, Ambrish Tiwari, Kalpana Luthra, S. K. Sharma, Archana Singh

**Affiliations:** 1 Department of Biochemistry, All India Institute of Medical Sciences, New Delhi, 110029 India; 2 Department of Biochemistry, All India Institute of Medical Sciences, New Delhi, 110029 India; 3 Department of Biochemistry, All India Institute of Medical Sciences, New Delhi, 110029 India; 4 Department of Medicine, All India Institute of Medical Sciences, New Delhi, 110029 India; 5 Department of Biochemistry, All India Institute of Medical Sciences, New Delhi, 110029 India

**Keywords:** Genetic association study, Tuberculosis

## Abstract

Status of Fok I VDR polymorphism along with vitamin D, Vitamin D receptor (VDR), and cathelicidin levels in Tuberculosis (TB) patients compared to household contacts and implication of these findings in susceptibility to TB is not known. 150 active TB patients, 150 household contacts and 150 healthy controls were recruited from North Indian population. Fok1 VDR polymorphism was studied by polymerase chain reaction- restriction fragment length polymorphism (PCR-RFLP).VDR mRNA and protein levels were studied using quantitative real time PCR (q rt PCR) and enzyme linked immunosorbent assay (ELISA) respectively. Cathelicidin and Vitamin D levels were measured using ELISA and chemiluminescence immunoassay (CLIA) respectively. Significant association was found between Fok1 polymorphism and susceptibility to TB (P < 0.0005). VDR mRNA, VDR protein and vitamin D levels were significantly lower in active TB group when compared to household contacts and healthy controls (P < 0.0001, 0.0001 and 0.0005 respectively). Cathelicidin levels were higher in active TB patients compared to other groups (P < 0.0001). Expression of VDR and cathelicidin was significantly higher among ‘FF’ genotypes of VDR (more active form of VDR) compared to ‘ff’ genotype (less active form of VDR). ‘f’ allele was associated with increased susceptibility to TB. Higher frequency of ‘F’ allele, increased VDR expression along with increased vitamin D levels in household contacts compared to active TB group might be responsible for protection against active TB.

## Introduction

Tuberculosis (TB) remains one of the most important causes of death from an infectious agent, with a latest World Health Organization (WHO) figures indicating an estimating 10.4 million new incident cases globally in 2017, out of which 26% incident cases are from India^1^. The main etiologic agent of tuberculosis is an intracellular slightly gram positive bacteria, *Mycobacterium tuberculosis* (M.TB.). This intracellular bacterium can evade the host immune system and can remain in the macrophages of the host in dormant stage until the immunity is suppressed. The question arises, why the infection remains in latent phase in some individuals and active in others? What are the host factors that play role in activation of the disease? It is seen that some household contacts of tuberculosis patient may or may not develop tuberculosis even after the long term exposure to M.TB.

There are various environmental and genetic factors which are responsible for susceptibility to TB. Two such factors are vitamin D and its nuclear vitamin D receptor (VDR). Vitamin D acts as an immunomodulatory molecule and plays a role in the innate immune response in individuals to certain infectious agents, M.TB^2^. Binding of the active form of Vitamin D to its nuclear receptor VDR leads to formation of an active transcriptional complex, which increases expression of a number of proteins. One such protein is cathelicidin (LL37) which is involved in killing of M.TB.

Upon M.TB. infection monocyte toll-like receptors (TLR1–TLR2) gets activated which results in the upregulation of VDR and the vitamin-D-activating enzyme 25-hydroxyvitamin D-1α-hydroxylase. 25-hydroxyvitamin D is bound to serum vitamin-D-binding protein while in circulation. It is converted to its active form 1, 25-dihydroxyvitamin D by mitochondrial 25-hydroxyvitamin D-1α-hydroxylase in macrophages and then binds to VDR and acts as a transcription factor leading to the induction of cathelicidin (LL37) expression and promotion of autophagy. Antimycobacterial action of cathelicidin and autophagy may then combine to enhance bacterial killing^3,4^.

Cathelicidins are family of proteins consisting of C- terminal cationic anti-microbial peptide (CAMP) domain that is activated by cleavage from N- terminal cathelin portion of propeptide. These proteins are stored in the granules of neutrophils from which it can be released at sites of microbial infection^5^. Various white blood cells (WBC) populations also express this peptide^6^. It has been shown that exogenous addition of cathelicidin or endogenous overexpression of cathelicidin in macrophages significantly reduces the intracellular survival of M.TB. relative to control cells^7^. However the role of vitamin D and its receptor in controlling growth of M.TB. *in vivo* is a complex process and is not fully understood.

There is much variability among the individuals in response to M.TB. infections, but it is not known why certain people develop TB when infected with M.TB. while others remain latently infected. Genetic susceptibility has been suggested as one of the most important explanations for individual risk for TB. Studies have suggested a role for host genetics in TB susceptibility with respect to VDR gene polymorphism^8–10^. The Vitamin D receptor (*VDR)* gene is located on the long (q) arm of chromosome 12 at position 13.11. After binding to its ligand, vitamin D, VDR forms a heterodimer with retinoid X receptor. This heterodimer binds to vitamin D response elements (VDREs) in the promoter region of vitamin D3 regulated genes, enabling the interaction of regulatory proteins thereby mediating transcription. Various VDR polymorphisms have been observed in association with TB, particularly in the Fok1 SNP in exon2, Bsm1 in intron 8 and Taq 1 in intron 9 but with inconclusive findings^9–13^. As the function of vitamin D at nuclear level is carried along with VDR, it is pertinent to study VDR polymorphism. The Fok1 polymorphism is one of the functional and important polymorphism of VDR gene.

Fok I polymorphism results in different translation initiation sites due to thymine (T) to cytosine (C) substitution in the first translation initiation codon ATG which generates long and short forms of *VDR*. In the *VDR* ‘ff’ variant, initiation of translation occurs at the first ATG site, giving rise to a full length *VDR* protein comprised of 427 amino acids. In the *VDR* ‘FF’ variant, translation begins at the second ATG site due to the substitution at first site, resulting in a truncated protein with 424 amino acids. It has also been reported that ‘FF’, the shorter form of VDR protein is the more active form than its full length protein, ‘ff’^14,15^. The Fok1 polymorphism thus may affect function of VDR resulting in altered efficiency of binding to vitamin D, which in turn may affect production of downstream molecules such as cathelicidin. Hence this polymorphism could be one of the factors influencing increased susceptibility to TB infection.

Studies so far have correlated low levels of vitamin D and VDR polymorphism independently with occurrence of TB. The association of functional VDR polymorphism (Fok1) on expression of VDR and cathelicidin levels in TB patients is still unknown. Also the levels of VDR and cathelicidin in household contacts of TB patients have not been studied. This study aimed to explore the role of vitamin D, VDR, its polymorphism and their association on production of cathelicidin *in vivo* in TB patients and their household contacts at levels of vitamin D prevalent in north Indian population.

## Results

### Study population

A total of 150 newly diagnosed active TB patients and 150 household contacts of active TB patients were recruited. 150 healthy individuals with no known history of TB were recruited as controls. For our study we included only pulmonary tuberculosis (only lung parenchyma involved) based on inclusion and exclusion criteria (discussed in methodology section). A case of pulmonary TB phenotype is described as: a clinically diagnosed case of TB affecting the lungs, having symptoms of fever or cough and sputum smear that showed acid-fast bacilli or culture positive for M.TB. or Gene Xpert positive. We excluded all patients with concurrent pulmonary–extrapulmonary disease. Therefore only one clinical phenotype as described above was studied in the present study. The mean age of active TB patients was 39.4 ± 8.1 years, household contacts was 35.4 ± 6.6 years and healthy controls was 32.1 ± 4.1 years. The demographic and clinical characteristics of study participants are shown in Table 1.

**Table 1:** Socio demographic and clinical characteristics of study participants.

Demographic characteristics	Active TB patients (n = 150)	Household contacts (n = 150)	Healthy controls(n = 150)
Age (Mean ± SD)	39.4 ± 8.1	35.4 ± 6.6	32.1 ± 4.1
Male (%)	68	73.86	87
Female (%)	32	26.13	13
Race	North Indian	North Indian	North Indian
Recruited in Winter (%)	20.6	22	12
Recruited in Spring (%)	32	26	37.3
Recruited in Summer (%)	38	43	38
Recruited in Autumn (%)	9.3	9	13.3
Smoking (%)	42.6	21.59	18
Non- vegetarian (%)	73.3	68.18	74
Vegetarian (%)	26.7	31.82	26
Indoor Occupation (%)	30	44.23	34
Outdoor Occupation (%)	70	55.77	65
Body Mass Index (Kg/m^2^) in mean ± SD	18.6 ± 4.4	24.8 ± 4.3	23.9 ± 3.2
Blood Glucose levels (mmol/L)	4.7 ± 1.6	5.2 ± 0.9	4.2 ± 0.9
Abnormal chest X ray (%)	96.36	None	None
*Gene expert (%)	94.5	Not done	Not done
*AFB positive smear (%)	78.66	Not done	Not done

*Gene Xpert positive: Positive for nucleic acid amplification (NAA) test for M.Tb. genome present in TB cases.

*AFB positive: Positive for acid fast bacilli staining.

### Significant association of Fok 1 VDR polymorphism with susceptibility to TB

**Figure 1:**
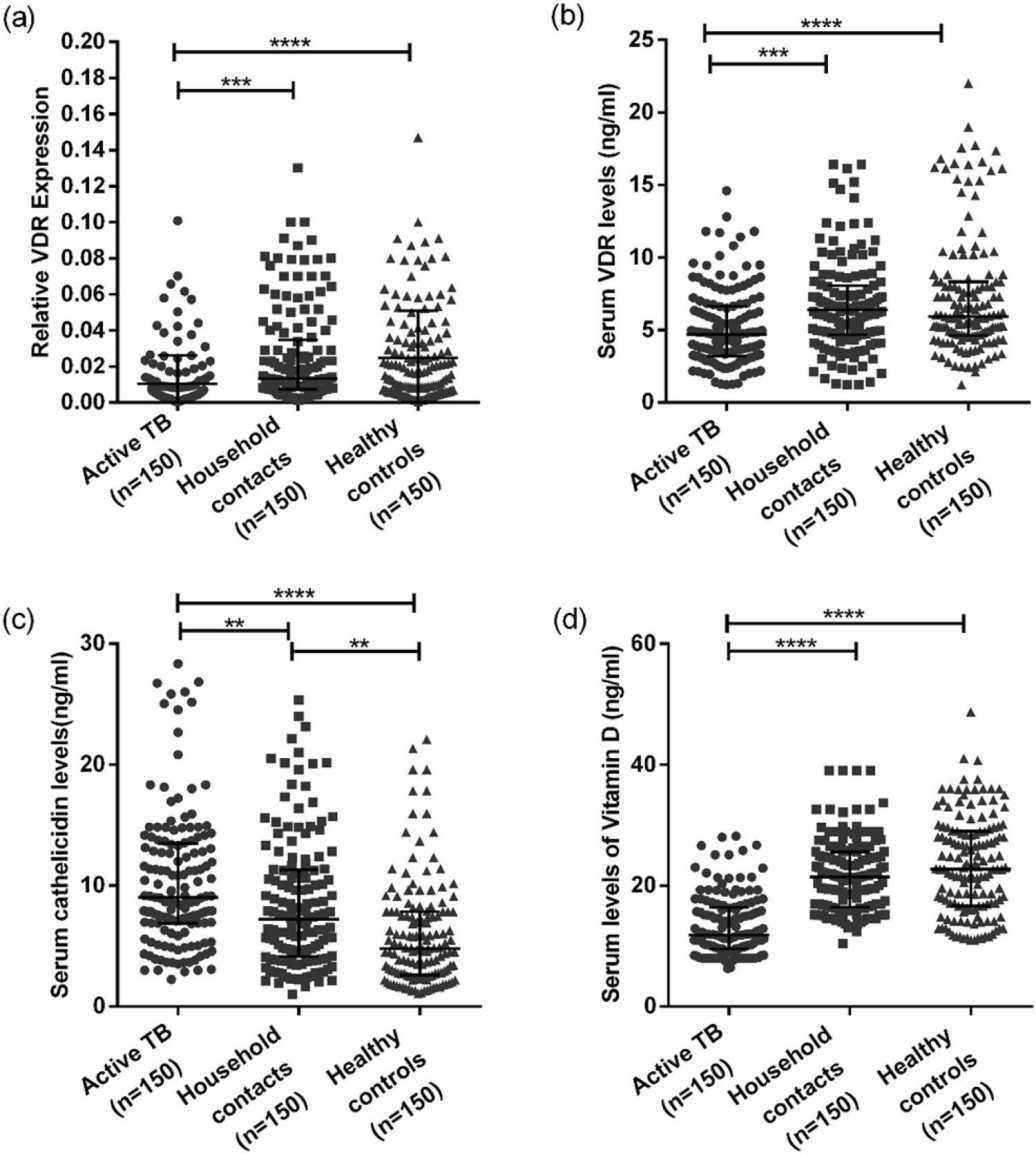
Low levels of vitamin D and its associated molecules active TB patients. 1a and 1b show the expression of VDR at mRNA and protein level respectively and (**c**,**d**) show the serum levels of cathelicidin and vitamin D in active TB patients, Household contacts and Controls. Data is represented as median ± standard deviation and each point represents individual sample value. Mann-Whitney U test was used for comparison of values between groups. One asterisk (*) indicates a p-value < 0.05; two asterisk (**) indicate a p-value < 0.01, three asterisk (***) indicate a p-value < 0.001 and four asterisk (****) indicate a p-value < 0.0001. Active TB = Naïve active pulmonary TB; Household contacts = contacts living with the pulmonary TB patients for minimum of 2 months with no sign and symptoms of TB; Control = Healthy controls with no history of TB.

The Fok 1 VDR polymorphic variant obtained by using polymerase chain reaction-restriction fragment length polymorphism (PCR-RFLP) using Fok1 restriction enzyme is shown in Fig. 1. The genotypic distribution was in Hardy-Weinberg equilibrium. The frequencies of Fok I genotypes at VDR locus showed a significant difference in the distribution of genotypes. ‘ff’ homozygous genotype was observed in 24.6% of the active TB patients, 9.3% of household contacts and 8.6% of healthy controls. The ‘Ff’ heterozygous genotype was observed in 38.6% of patients as compared to 36.6% of household contacts and 34.0% of controls. ‘FF’ homozygous genotype was present in 36.6% of patients as compared to 54% of household contacts and 57.3% of controls. The frequency of ‘ff’ (long form of VDR) genotype was higher in patient group and that of ‘FF’ genotype was higher in control group and the differences were statistically significant (χ^2^ value of 25.22, df of 4 and p value = 0.0001). The frequency of ‘F’ allele was found to be 56% in active patients and 72.3% in household contacts as compared to 74.3% in healthy controls. The frequency of ‘f’ allele was found to be 44% in active patients, 27.7% in household contacts and 25.7% in healthy controls. The difference was found to be significant suggestive of an association of Fok1 VDR polymorphism with susceptibility to TB (Tables 2 and 3). Out of 150 patients, 118 were found to be sputum positive with variable sputum grade, 25 being +1, 81 being +2 and 12 being + 3. The frequency of genotype in different sputum grades (+1, +2 and + 3) were also analysed and found to be associative with smear grades (Table 4).

**Table 2:** Frequency of *Fok1 VDR* genotypes and alleles in Active TB patients, household contacts and controls.

Polymorphism	Genotype	Patients (150) n (%)	Household contacts (150) n (%)	Controls (150) n (%)	χ2 value	p-value
Fok1 VDR (T/C)	FF	55 (36.6)	81 (54)	86 (57.3)	25.22	0.0001****
	Ff	58 (38.6)	55 (36.6)	51 (34.5)		
	ff	37 (24.6)	14 (9.3)	13 (8.6)		
**Allele**	**Patients (%)**	**Controls (%)**		**Household contacts (%)**		**p- value**
F	56	72.3		74.3		0.01*
f	44	27.7		25.7		

Active TB = Naïve active pulmonary TB; Household contacts = contacts living with the pulmonary TB patients for minimum of 2 months with no sign and symptoms of TB; Control = Healthy controls with no history of TB. Chi square test was used for genotypic analysis. One asterisk (*) indicates a p-value < 0.05; two asterisk (**) indicate a p-value < 0.01, three asterisk (***) indicate a p-value < 0.001 and four asterisk (****) indicate a p-value < 0.0001.

**Table 3:** Odds ratio (OR) and confidence interval (CI) of *Fok1 VDR* genotypes and alleles in Active TB patients and household contacts compared to controls.

Fok 1	Patients (%)	Controls (%)	OR	CI	P-value
**Active pulmonary TB patients vs Healthy controls**					
**Genotypes**					
FF	36.6	57.3	0.41	0.21–0.74	0.004**
Ff	38.6	34	0.76	0.49–1.79	0.47
ff	24.6	8.6	9.73	3.96–29.15	0.0001****
**Alleles**					
F	56	74.3	0.31	0.17–0.60	0.0003***
f	44	25.7	3.14	1.72–4.96	0.0003***
**Fok 1**	**Household contacts (%)**	**Controls (%)**	**OR**	**CI**	**P-value**
**Household contacts vs healthy controls**					
**Genotypes**					
FF	54	57.3	0.32	0.19–0.65	0.0005***
Ff	36.6	34	2.07	1.13–3.62	0.01*
ff	9.3	8.6	3.1	0.92–10.19	0.05*
**Alleles**					
F	72.3	74.3	0.47	0.32–0.77	0.005**
f	27.7	25.7	2.54	1.30–4.59	0.005**

Active TB = Naïve active pulmonary TB; Household contacts = contacts living with the pulmonary TB patients for minimum of 2 months with no sign and symptoms of TB; Control = Healthy controls with no history of TB. Odds ratios (ORs) and 95% confidence intervals (CIs) were calculated to quantitatively assess the strength of association between the polymorphisms and TB. One asterisk (*) indicates a p-value < 0.05; two asterisk (**) indicate a p-value < 0.01, three asterisk (***) indicate a p-value < 0.001 and four asterisk (****) indicate a p-value < 0.0001.

**Table 4:** Frequency of *Fok1 VDR* genotypes in different sputum grade active TB patients,

Polymorphism	Genotype	+1 sputum smear (N)	+2 sputum smear (N)	+3 sputum smear (N)	χ2 value	p-value
Fok1VDR (T/C)	FF	16	16	3	24.42	0.0001****
	Ff	5	42	2		
	ff	4	23	7		

### Lower mRNA expression of VDR in active TB patients as compared to household contacts and healthy controls

VDR mRNA expression in WBCs of active TB patients and household contacts was measured by quantitative real-time PCR (q rt PCR) and was compared to healthy controls. Comparison of VDR expression was made by determining the threshold cycle (C_t_) values and was then normalized with Glyceraldehyde 3-phosphate dehydrogenase (GAPDH). We have observed that active TB patients had significantly lower expression of VDR mRNAs compared to household contacts and healthy controls (P < 0.001 and 0.0001 respectively) (Fig. 1a).

### Lower serum levels of VDR and higher levels of cathelicidin (LL37) in active TB patients compared to household contacts and healthy controls

The serum levels of vitamin D receptor and cathelicidin in active TB patients, household contacts and control group were measured by sandwich ELISA. A significant difference in the serum levels of vitamin D receptor and cathelicidin was observed among all the three groups with p < 0.0001. The levels of VDR were lower in patient groups (5.13 ± 2.6 ng/ml) as compared to household contacts (6.69 ± 3.17 ng/ml) and healthy controls (7.30 ± 4.15 ng/ml) with p < 0.001 and 0.0001 respectively. The levels of VDR in household contacts and healthy controls were comparable. The serum levels of cathelicidin was significantly higher in active TB group (10.36 ± 5.62 ng/ml) as compared to household contacts (8.48 ± 5.45 ng/ml) and healthy controls (5.99 ± 4.44 ng/ml) with p < 0.01 and 0.0001 respectively. Unlike VDR, a significant difference was observed in the cathelicidin levels between household contacts and healthy controls (p < 0.01) (Fig. 1b,c).

### Lower serum levels of Vitamin D in active TB patients compared to household contacts and healthy controls

Serum vitamin D levels varied among all the groups with lowest levels seen in active TB patient group (13.28 ± 5.0 ng/ml) as compared to household contacts (21.62 ± 5.99 ng/ml), and healthy controls (23.31 ± 8.2 ng/ml). The difference was statistically significant (p < 0.0001). The levels were comparable between household contacts and healthy controls (Fig. 1d).

### Association of genotype with VDR and cathelicidin expression

**Figure 2:**
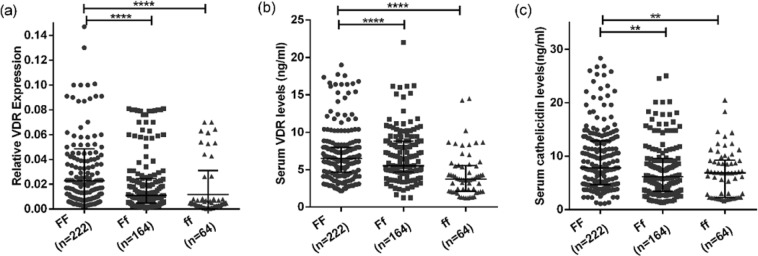
Association of genotype with VDR and cathelicidin levels irrespective of study groups: (**a,b**) shows the expression of VDR at mRNA and protein levels respectively with respect to different genotypes. (**c**) shows the level of cathelicidin with respect to different genotypes. Mann-Whitney U test was used for comparison of values between groups. One asterisk (*) indicates a p-value < 0.05; two asterisk (**) indicate a p-value < 0.01, three asterisk (***) indicate a p-value < 0.001 and four asterisk (****) indicate a p-value < 0.0001. Active TB = Naïve active pulmonary TB; Household contacts = contacts living with the pulmonary TB patients for minimum of 2 months with no sign and symptoms of TB; Control = Healthy controls with no history of TB.

A comparison of the expression of VDR among different genotypes of VDR irrespective of the study groups showed that samples with ‘FF’ genotype (more active form of VDR) have higher expression of VDR at both mRNA and serum protein level as compared to ‘ff’ genotype (less active form of VDR) (p < 0.0001 and 0.0001 respectively) (Fig. 2a, 2b). The serum levels of cathelicidin were found to be significantly higher in ‘FF’ genotype as compared to ‘Ff’ and ‘ff’ genotype with (p < 0.01) (Fig. 2c).

**Figure 3:**
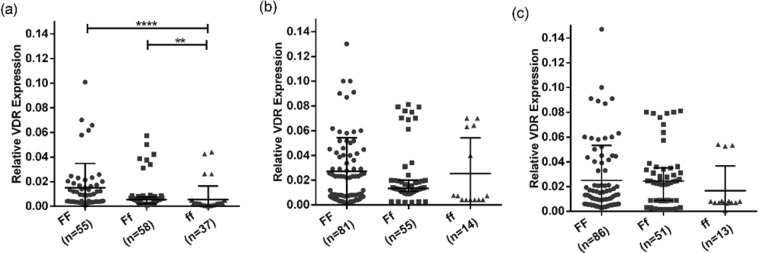
Association of genotype with mRNA expression of VDR and cathelicidin levels in different study groups: (**a**–**c**) shows the expression of VDR mRNA in active TB patients, household contacts and healthy controls respectively with respect to different genotypes. Mann-Whitney U test was used for comparison of values between groups. One asterisk (*) indicates a p-value < 0.05; two asterisk (**) indicate a p-value < 0.01, three asterisk (***) indicate a p-value < 0.001 and four asterisk (****) indicate a p-value < 0.0001. Active TB = Naïve active pulmonary TB; Household contacts = contacts living with the pulmonary TB patients for minimum of 2 months with no sign and symptoms of TB; Control = Healthy controls with no history of TB.

**Figure 4:**
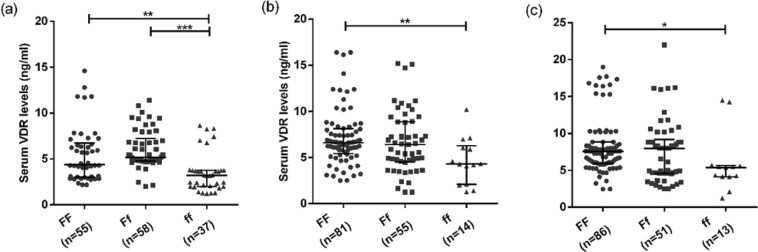
Association of genotype with serum VDR levels in different study groups: (**a**–**c**) shows the level of VDR protein in active TB patients, household contacts and healthy controls respectively with respect to different genotypes. Mann-Whitney U test was used for comparison of values between groups. One asterisk (*) indicates a p-value < 0.05; two asterisk (**) indicate a p-value < 0.01, three asterisk (***) indicate a p-value < 0.001 and four asterisk (****) indicate a p-value < 0.0001. Active TB = Naïve active pulmonary TB; Household contacts = contacts living with the pulmonary TB patients for minimum of 2 months with no sign and symptoms of TB; Control = Healthy controls with no history of TB.

**Figure 5:**
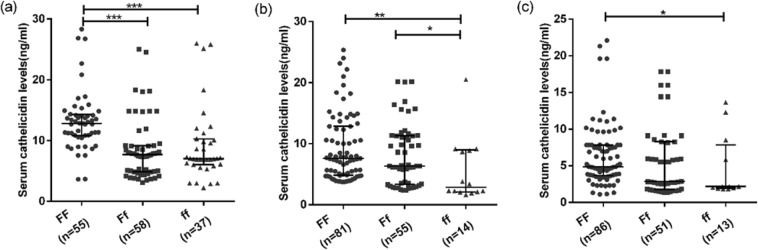
Association of genotype with serum cathelicidin levels in different study groups: (**a**–**c**) shows the level of cathelicidin in active TB patients, household contacts and healthy controls respectively with respect to different genotypes. Mann-Whitney U test was used for comparison of values between groups. One asterisk (*) indicates a p-value < 0.05; two asterisk (**) indicate a p-value < 0.01, three asterisk (***) indicate a p-value < 0.001 and four asterisk (****) indicate a p-value < 0.0001. Active TB = Naïve active pulmonary TB; Household contacts = contacts living with the pulmonary TB patients for minimum of 2 months with no sign and symptoms of TB; Control = Healthy controls with no history of TB.

Comparison of the expression levels of both VDR and cathelicidin among different genotypes within each of the groups showed a significant difference between ‘FF’ and ‘ff’ genotypes, with individuals having ‘FF’ genotypes have higher levels of VDR and cathelicidin as compared to individuals having ‘ff’ genotype (Figs 3–5).

## Discussion

Tuberculosis burden could be reduced with decreasing incidence of new infection and effective therapy of newly diagnosed cases. Household contacts are at higher risk of developing latent or active TB or complete elimination of TB depending upon the host immune response. First line of defense against TB is innate immunity comprises of effector functions of innate immune cells. Therefore host factors affecting innate immunity could affect outcome of the infection. So, with this background the research question was to study host factors affecting innate immunity in pulmonary TB. Vitamin D is an immunomodulatory molecule; along with VDR it induces expression of various proteins like cathelicidin which is an important antimicrobial agent for intracellular pathogens. However VDR polymorphism could affect synthesis and functionality of VDR protein, affecting cathelicidin. Therefore this study intended to assess vitamin D levels along with effect of VDR polymorphism on disease outcome.

The present study showed significant association of Fok1 SNP (f allele) with the susceptibility to TB. The findings are in concordance with one of the study in Chinese Han population showing association of ‘ff’ genotype with susceptibility to TB^9^. Similar findings were observed in a north Indian population showing significant association of Fok1 VDR polymorphism with leprosy^16^. However, in other studies from Egypt and India, no significant association was observed for VDR Fok1 polymorphism with susceptibility to TB^10,17^. Interestingly, in one of the study in south Indian population, the wild type genotype (‘FF’) was found to be associated with susceptibility to TB^11^, whereas in the present study, ‘ff’ genotype (homozygous mutant) was found to be associated with susceptibility to TB. In a meta analysis for VDR Fok1 polymorphism in TB, several other VDR polymorphism like Bsm1, Taq1, Apa1 and Fok1 has also been studied showing Fok1 association with susceptibility to TB in east Asian population^18^. The diversity of genetic background in different populations may contribute to variable genotype frequencies of VDR polymorphisms and its inconsistent association with TB development.

Further, the expression of VDR at mRNA levels was found to be significantly lower in WBCs of active TB patients as compared to household contacts and healthy controls. In an earlier study conducted in pulmonary TB patients, expression of VDR mRNA was found to be significantly higher in M.TB. stimulated macrophage cultures compared to unstimulated cultures^19^. The results were in accordance with another study in which lower expression of VDR was observed in leprosy patients as compared to controls^20^. Based on observations made in present study, it was hypothesized that M.TB. may alter the expression of some of the genes like VDR in order to evade the host immune system, however the exact mechanism needs to be studied. Another study based on microarray has shown a 3.3 fold downregulation in VDR expression in cells upon infection with M.TB.^21^. Similar to VDR mRNA expression, VDR protein levels were found to be lower in active TB group as compared to household contacts and healthy controls. Therefore lower expression of VDR in the active TB patient group in the present study may be due to an attempt by the bacteria to downregulate the expression of VDR for its own advantage, so that cathelicidin levels would be hampered and the bacteria can survive in the host. This is the first study wherein VDR expression in household contacts was assessed and found to have higher expression of VDR as compared to TB patients. This might be due to the sufficient vitamin D levels present in the household contacts. However, the exact mechanism behind the same needs to be studied further.

As VDR is regulated by its ligand, vitamin D, its levels were measured in all the groups. A lower vitamin D level in active TB patients as compared to both household contacts and normal healthy controls was observed. These findings are in concordance with studies showing lower serum levels of vitamin D in tuberculosis patients^22,23^. Low levels of vitamin D in active TB patient group may be one of the factors leading to decreased expression of VDR and hence increased susceptibility to TB.

The serum levels of antimicrobial peptide, cathelicidin was also assessed and found to be higher in active TB patients as compared to both household contacts and healthy controls. Present findings are in accordance with one of the study in which active TB patient group had higher levels of cathelicidin as compared to healthy controls^24,25^. Similarly, increase in cathelicidin level in active disease is also documented in an *in vitro* model where it was shown that multiplication of bacteria stimulated TLR by a pathogen-derived ligand, which acted as a trigger for upregulation of cathelicidin to exert its anti-microbial activity^3^. Even though vitamin D insufficiency was observed in active TB patients, cathelicidin levels were found to be higher. Therefore, localized or intracellular cathelicidin concentrations needs to be evaluated for better understanding of interaction between vitamin D status and localized LL-37 responses within immune cells and at localized immunologic barrier sites. This finding can be supported by a study where healthy volunteers who received vitamin D supplementation showed correlation between serum 25(OH)D concentrations and cathelicidin expression in primary *ex vivo* cultures of human monocytes, whereas systemic concentrations of cathelicidin did not correlate with serum vitamin D concentrations^26^. In the present study, higher levels of cathelicidin was observed in household contacts compared to healthy controls. Another study has shown that cathelicidin antimicrobial peptide (CAMP) gene expression was significantly augmented in progressive TB, whereas in latent TB it was similar to the control group^27^. Based upon above findings, it could be hypothesized that household contacts of active TB patients might have some M.TB. infection which could be the reason for increased level of cathelicidin compared to healthy controls, sufficient to curtail overwhelming multiplication, though lower than active TB patients.

Levels of VDR and cathelicidin in active TB patients based on their sputum grade was also compared. No significant difference in the levels of either VDR and cathelicidin in different sputum AFB grades of patients (data not shown) was found. However, the same needs to be validated in larger sample size as in our study, out of 150 patients, we had 118 sputum smear positive patients, out of which only 12 were found to be in + 3 grade.

Till date, no study has been conducted to assess the association of functional Fok1 VDR genotypes with its downstream molecules like vitamin D receptor and cathelicidin. Here in for the first time it was observed that samples with ‘FF’ genotype (more active form of VDR) have higher expression of VDR compared to ‘ff’ (less active form of VDR) genotype, suggesting the association of ‘FF’ VDR genotype with higher expression of VDR might have protective role against TB. However results need to be validated in a larger sample population.

Since one of the effector molecule in vitamin D pathway is cathelicidin, which is responsible for killing of the bacteria, association between Fok1 genotypes and cathelicidin levels were assessed. Higher levels of cathelicidin were found to be associated with ‘FF’ (active form of VDR) genotype suggesting their plausibly combined protective role against the disease. Subgroup analysis for levels of VDR and cathelicidin among different grades of sputum AFB positive patients with respect to different genotypes was done to assess any association of genotype and levels of VDR and cathelicidin with respect to disease severity. However, no significant association was observed suggesting that ‘ff’ genotype is associated with lower levels of VDR and cathelicidin but it did not correlated with disease severity. However, apart from the association of VDR genotypes with VDR protein and cathelicidin, several other environmental and host factors might also play an important role in susceptibility to TB.

To conclude, the ‘ff’ genotype of VDR Fok1 polymorphism was found to be significantly associated with susceptibility to TB. High expression levels of VDR and vitamin D in household contacts of TB patients are implicative of the protective role of vitamin D against activation of TB and should be studied further to be considered as a preventive strategy against development of active TB. Also, cathelicidin can be used as biomarker for diagnosing TB amongst household contacts, however, the same needs to be validated in larger sample size. The limitation of the study is that household contacts have not been followed up for development of active disease. Also, localized levels of cathelicidin and VDR needs to be assessed in BAL (bronchoalveolar lavage) samples of pulmonary TB patients to get a more mechanistic interpretation. Further studies can be done to follow up this high risk group for assessing the change in the vitamin D related host factors with development of active disease to use in preventive strategy.

## Materials and Methods

### Study Participants

The present cross sectional study was carried out in the Department of Biochemistry at All India Institute of medical Sciences (AIIMS). Active TB patients (Newly diagnosed laboratory confirmed cases of pulmonary tuberculosis) who had not received more than 2 weeks of antitubercular treatment (ATT) were recruited from directly observed treatment (DOTS) Centre, AIIMS, New Delhi, India based on inclusion and exclusion criteria. A case of pulmonary TB was defined as a clinically diagnosed case of TB affecting the lungs, having symptoms of fever or cough and sputum smear that showed acid-fast bacilli or culture positive for M.TB. or Gene Xpert. Exclusion criteria included patients with extra-pulmonary and drug resistant tuberculosis, HIV, diabetes, hypertension, significant organ dysfunction of heart, liver and kidney, pregnant or lactating women. Additional exclusion criteria were known hypersensitivity to ATT, seizure disorder, abnormal hematologic function, inflammation like autoimmune disease, atopic dermatitis or any other uncontrolled concurrent illness. Patients with prior anti-microbial drug treatment of TB for longer than two weeks and any history of alcohol and drug abuse were also excluded.

Household contacts of TB patients aged between 18–60 years who had spent atleast 6 hours per day for atleast 2 months prior with the TB patient before start of ATT with no clinical evidence of TB were recruited. The exclusion criteria were same as for the active TB patients. Mantoux test was done in household contacts. Mantoux negative individuals were directly recruited under household contacts group. Mantoux positive individuals were followed up by sputum test or chest X-ray. Individuals negative for these tests were recruited under household contacts. Individuals with positive test were further investigated for active disease. Individuals with altered clinical parameters like Hemogram, liver function test, kidney function test, blood glucose, etc and those who were taking vitamin D supplementation were excluded from the study. Normal healthy controls were recruited from the general population with no history of known contacts with TB patients and no clinical signs and symptoms of tuberculosis and no history of any other chronic disorder or inflammation. 150 active TB patients, 150 household contacts and 150 healthy controls were recruited from North Indian population for the study after obtaining written informed consent.

The study was conducted adopting the ethical principles stated in the latest version of Helsinki Declaration as well as the applicable guidelines for good clinical practice (GCP). Ethical approval was obtained from Institutional Ethics Committee of All India Institute of Medical Sciences, New Delhi (Ref NO: IEC/NP-299/07.08.2015, RP-03/2015). The demographic profile and clinical characteristics of study participants is shown in Table 1

### Sample collection and storage

6 mL of peripheral venous blood was collected from patients and controls. The blood specimen was given a personal identifier number that was used to link and maintain the biological information derived. 4 mL blood was for serum separation and was stored at −70 °C for further analysis. 2 mL blood was collected in EDTA vials for the separation of DNA and RNA for genomic and expression studies.

### Genotyping for Fok1 VDR Single Nucleotide Polymorphism (SNP)

Genomic DNA was isolated from blood using Relia prep Blood gDNA Miniprep system (A5081), Promega. The Fok1 VDR SNP was performed by PCR-RFLP. PCR was done using Thermo Scientific, #K0171 master mix to amplify the desired sequence of VDR using specific primers. The sequence of forward and reverse primers were as follows:

Forward-(5′-AGCTGGCCCTGGCACTGACTCTGCTCT-3′)

Reverse- (5′-ATGGAAACACCTTGCTTCTTCTCCCTC-3′)

with cycling parameters as follows: Denaturation at 95 °C for 5 min, 35 cycles at 95 °C for 30 s, 65.8 °C for 30 s and 72 °C for 60 s and one final cycle of extension at 72 °C for 5 min. The PCR product (10 µl) of 280 bp was then digested with 1.0 unit of Fok I restriction enzyme (#FD2144, Thermo Scientific) and incubated at 37 °C for 15 min. Genotypic patterns were determined by resolving the digested products on a 2% agarose gel (Figure S1).

### Quantitative PCR (qPCR) for expression study

RNA isolation was done from whole blood using TRIzol method and cDNA was synthesized using Verso cDNA synthesis kit (AB1453A, Thermo). Real time detection of VDR was done using above mentioned specific primers of VDR mRNA using 2X Maxima SYBR Green qPCR master mix (Thermo scientific). The PCR amplification was performed in a 10 μl reaction volume with cycling parameters as follows: Denaturation at 95 °C for 5 min, 35 cycles at 95 °C for 30 s, 64 °C for 45 s and 72 °C for 30 s and one final cycle of extension at 72 °C for 5 min. GAPDH was used as a reference gene. A melt curve analysis was performed after every reaction to confirm the specific amplification of products.

### Estimation of serum levels of Vitamin D

Serum levels of Vitamin D were estimated for all samples using automated chemiluminiscence based immunoassay system (VITROS ECi, Johnson and Johnson Ortho Clinical Diagnostics). Competitive immunoassay was used where the light signals read by the system is inversely proportional to the concentration of 25-OH vitamin D present in the sample.

### Estimation of serum levels of VDR and cathelicidin

Serum levels of VDR was measured using a Human VDR Sandwich ELISA kit (SEA475Hu, USCN, Life Science Inc., Wuhan, Hubei).

Serum levels of cathelicidin was measured using Human LL-37 Sandwich ELISA kit, HK321 (Hycult Biotech, Netherlands).

### Statistical analysis

All statistical analyses were performed on GraphPad Prism 6 (GraphPad Software Inc., San Diego, CA, USA). Non-parametric statistical analyses were performed throughout the study. Kruskal Wallis and Mann-Whitney U test was used for comparison of variables between three and two groups respectively. The polymorphic variants were analysed by chi-square test with values predicted by the Hardy Weinberg equilibrium model. Odds ratios (ORs) and 95% confidence intervals (CIs) were calculated to quantitatively assess the strength of association between the polymorphisms and TB. Values were expressed as median ± standard deviation. A p-value of less than 0.05 was considered significant.

## Supplementary information

Supplementary information 
