# Supplementary material for: Association of Fok1 VDR polymorphism with Vitamin D and its associated molecules in pulmonary tuberculosis patients and their household contacts
Source: Sci Rep. 2019 Oct 24;9:15251. doi: 10.1038/s41598-019-51803-8 (PMC6813333; doi:10.1038/s41598-019-51803-8)
Supplement: Supplementary file 1 — Supplementary information [file 41598_2019_51803_MOESM1_ESM.pdf]

TITLE PAGE

TITLE: Association of Fok1 VDR polymorphism with Vitamin D and its associated molecules in pulmonary tuberculosis patients and their household contacts

AUTHORS: Sudhasini Panda[1], Ambrish Tiwari[2], Kalpana Luthra[3], S.K. Sharma[4], Archana Singh[5]

AUTHORS AFFLIATION:

1) Sudhasini Panda (PhD), Department of Biochemistry , All India Institute of Medical Sciences, New Delhi -110029, India.
2) Ambrish Tiwari (MD), Department of Biochemistry , All India Institute of Medical Sciences, New Delhi -110029, India.
3) Kalpana Luthra (PhD), Department of Biochemistry , All India Institute of Medical Sciences, New Delhi -110029, India.
4) S.K. Sharma (MD), Department of Medicine , All India Institute of Medical Sciences, New Delhi -110029, India.
5) Archana Singh (MD), Department of Biochemistry, All India Institute of Medical Sciences, New Delhi -110029, India.

INSTITUTION: Department of Biochemistry, Department of Medicine, All India Institute of Medical Sciences, New Delhi-110029

Corresponding Author: Dr. Archana Singh, Assistant Professor

Department of Biochemistry

All India institute of Medical sciences

Delhi -110029, India.

e-mail : archanasinghaiims@gmail.com

Phone: (0) 986845025

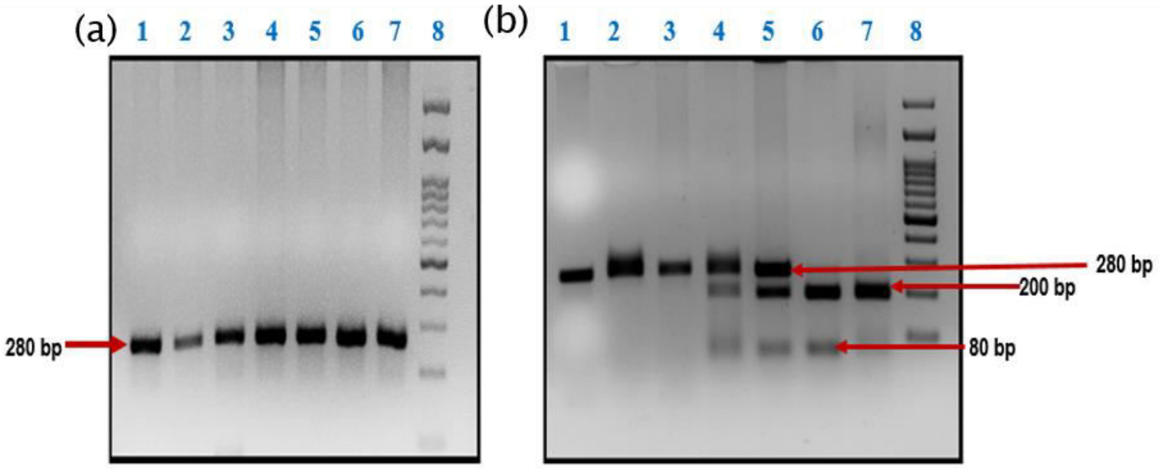

Figure S1: figure S1a shows 1.5% agarose gel showing PCR amplified products of *Fok1 VDR*

1 to 8 – lanes (L); L1 to L7: 280 bp PCR product; L8: 100bp DNA ladder

Figure S1b shows Restriction digestion products on 2% agarose gel for *Fok1 VDR SNP*

L1- NTC; L2, L3: FF genotype; L4, L5: Ff genotype; L6, L7: ff genotype; L8: 100 bp DNA ladder

Note: The above gel pictures are taken from two different gel. First gel image shows the bp PCR product of VDR gene of representative samples.

Second gel picture shows the restriction digested product of representative samples using FOK1 Fast digest enzyme

No cropping has been done in the gel image.
